# Host-Induced Silencing of FMRFamide-Like Peptide Genes, *flp-1* and *flp-12*, in Rice Impairs Reproductive Fitness of the Root-Knot Nematode *Meloidogyne graminicola*

**DOI:** 10.3389/fpls.2020.00894

**Published:** 2020-07-17

**Authors:** Alkesh Hada, Chanchal Kumari, Victor Phani, Divya Singh, Viswanathan Chinnusamy, Uma Rao

**Affiliations:** ^1^ Division of Nematology, ICAR-Indian Agricultural Research Institute, New Delhi, India; ^2^ Department of Agricultural Entomology, College of Agriculture, Uttar Banga Krishi Viswavidyalaya, Dakshin Dinajpur, India; ^3^ Division of Plant Physiology, ICAR—Indian Agricultural Research Institute, New Delhi, India

**Keywords:** root-knot nematodes, FMRFamide-like peptide, RNAi, *Oryza sativa*, transgenics

## Abstract

Rice (*Oryza sativa* L.) is one of the major staple food crops of the world. The productivity of rice is considerably affected by the root-knot nematode, *Meloidogyne graminicola*. Modern nematode management strategies targeting the physiological processes have established the potency of use of neuromotor genes for their management. Here, we explored the utility of two FMRFamide like peptide coding genes, *Mg-flp-1* and *Mg-flp-12* of *M. graminicola* for its management through host-induced gene silencing (HIGS) using *Agrobacterium-*mediated transformation of rice. The presence and integration of hairpin RNA (hpRNA) constructs in transgenic lines were confirmed by PCR, qRT-PCR, and Southern and Northern hybridization. Transgenic plants were evaluated against *M. graminicola*, where phenotypic effect of HIGS was pronounced with reduction in galling by 20–48% in the transgenic plants. This also led to significant decrease in total number of endoparasites by 31–50% for *Mg-flp*-*1* and 34–51% for *Mg-flp-12* transgenics. Likewise, number of egg masses per plant and eggs per egg mass also declined significantly in the transgenics, ultimately affecting the multiplication factor, when compared to the wild type plants. This study establishes the effectiveness of the two *M. graminicola flp* genes for its management and also for gene pyramiding.

## Introduction

Rice (*Oryza sativa* L.) is the staple cereal “global grain” constituting everyday meal of more than three billion people around the world. Although rice is popularly grown across different parts of the world, the tropical parts of Asia and South-East Asian countries produce approximately 90% of the global rice output (Mantelin et al., 2017). In the present era of modern farming, plant-parasitic nematodes (PPNs) pose a major threat to the agricultural food production and rice is also attacked by a wide array of nematodes (Prasad et al., 1992; Jones et al., 2013). Among the major PPN species attacking rice, the root-knot nematode (RKN) *Meloidogyne graminicola* Golden and Birchfield, 1965 alone has been reported to inflict up to 50% yield loss under different conditions (Lorenzana et al., 1998; Bridge et al., 2005). This economically important RKN species is widespread in almost all the rice growing areas in the world, and has been well documented to cause extensive yield and quality losses (Dutta et al., 2012; Kyndt et al., 2012). The infective pre-parasitic second-stage juveniles (J2s) of *M. graminicola* enter the roots and develop permanent feeding cells (giant cells) resulting in typical hook shaped galls (De Waele and Elsen, 2007).

Various strategies are used for management of PPNs, and the traditional practices are mostly relied on the use of cultural/physical methods and chemotherapeutics. However, due to the adverse effects of most of the nematicidal chemicals on environment, non-target organisms and human health, they were either discontinued or restricted for use in agricultural fields (Bridge et al., 2005; De Waele et al., 2013). Hence, there has been a continuous demand for development of environmentally benign target-specific nematode management approach, and genetic engineering based techniques have gained promising popularity in this regard (Roderick et al., 2018; Dutta et al., 2019). Genetic improvement of rice against PPNs through breeding programs faces a major challenge due to scant availability of suitable resistant sources (Soriano et al., 1999; Cabasan et al., 2012). Considerable natural resistance against *M*. *graminicola* has been reported in *Oryza glaberrima* and *O*. *longistaminata*, but limited resistant source has been reported in *O. sativa* (Soriano et al., 1999; Cabasan et al., 2012; Kumari et al., 2016; Kumari et al., 2017; Hatzade et al., 2020). The recent advancements in PPN genomics and transcriptomics have enabled us to identify the propitious molecular targets in nematodes that can be exploited for their management and also for drug designing (Danchin et al., 2013; Taylor et al., 2013; Shivakumara et al., 2019). The availability of draft genome can also offer a supportive platform to identify and validate the candidate genes responsible for rice–*M. graminicola* interaction (Somvanshi et al., 2018).

Nematode neuropeptides play a key role in controlling and modulating the physiological processes like host recognition, navigation, infection, secretion, reproduction etc., and have been proved to be potential drug targets (Maule et al., 2002; Kimber et al., 2007; Warnock et al., 2017). The FMRFamide-like peptides (FLPs) constitute a large and diverse group of neuropeptides in nematodes, governing the basic behavioral functions by coupling to the G-protein coupled receptors (GPCRs) (McVeigh et al., 2006; Atkinson et al., 2013). Similar results have also been reported due to silencing of *flp* genes that resulted in locomotory defects, reduced penetration, aberrant behavior and reduced reproduction in *Meloidogyne incognita* and *Globodera pallida* (Dalzell et al., 2010; Dong et al., 2013; Papolu et al., 2013; Banakar et al., 2015). However, scant knowledge is available to date with respect to performance of the *flp* gene repertoire against *M. graminicola* in rice through HIGS. Of late, HIGS has emerged as an effective and successful strategy to silence the genes in plant-parasitic nematodes for functional validation (Fairbairn et al., 2007; Danchin et al., 2013). In the present study, we have selected two *M. graminicola* FLP coding genes, *Mg-flp-1* and *Mg-flp-12*, for evaluation based on their reported effect on juvenile penetration and infectivity in rice. Previously, Rao et al. (2013) found that the translated sequence of *Mg-flp-12* putatively contains a N-terminal secretion signal peptide indicating its involvement in extra cellular signal transduction functioning like G protein coupled receptor (GO: 0004930); and similar finding was noted for *Mg-flp-1*, which contains the highly conserved LFRGR motif. These observations putatively indicated the potential of the genes as molecular target(s) against *M. graminicola*. Subsequently, Kumari et al. (2017) characterized nine *flp* genes from *M. graminicola* including *flp-1* and *flp-12*, and *in vitro* silencing of the said genes resulted in significantly reduced penetration of J2s and their infection potential in rice. Additionally, Masler (2008) showed that disruption of *flp-1* and *flp-12* results in various neuromuscular dysfunctions in other nematodes, and *flp-12* has also been proved to be a potential target against pine wood nematode, *Bursaphelenchus xylophilus* (Huang et al., 2010). Hence, to explore the potential of *flp-1* and *flp-12* as molecular targets, transgenic rice plants were developed expressing the dsRNA constructs of the two genes, and their effectiveness were evaluated against *M. graminicola* using soil-less Pluronic media and soil system. Thus, the present study strengthens our knowledge on the effect of *M. graminicola flp-1* and *flp-12* in nematode reproduction and plant parasitism potential, when applied through HIGS in rice.

## Materials and Methods

### Nematode Culturing

Pure culture of an Indian isolate of *M. graminicola* Golden and Birchfield, 1965 was maintained on rice (*O. sativa* cv. PB 1121) in a glasshouse at ICAR–Indian Agricultural Research Institute, New Delhi, India. Galls were handpicked from washed infected roots, and infective second-stage juveniles (J2s) were hatched using modified Baermann’s assembly (Whitehead and Hemming, 1965). The freshly hatched J2s were used for all the experiments.

### Preparation of Hairpin RNA (hpRNA) Constructs of Target Genes and Generation of Transgenic Plants

Previously, *flp-1* and *flp-12* were reported from *M. graminicola* by Rao et al. (2013) and subsequently Kumari et al. (2017) performed their molecular characterization and showed *in vitro* silencing effect of the genes on *M. graminicola* against rice. The partial sequence of *Mg-flp*-*1* (214 bp) and *Mg-flp*-*12* (299 bp) were then subjected to BlastN search against GenBank nonredundant (nr) databank and *M. graminicola* genome database (Altschul et al., 1990). Further, off-target sites in the targeted dsRNA were investigated at http://dsCheck.RNAi.jp/ for their presence, if any (Naito et al., 2005).

RNAi Gateway vector ph7GWIWG2(II) was obtained from VIB-UGent Center for Plant Systems Biology, Ghent University, Ghent, Belgium. Partial sequence of *Mg-flp*-*1* (214 bp), *Mg-flp*-*12* (299 bp) and an unrelated gene *gfp* (375 bp; green fluorescent protein used as non-native negative control) were PCR amplified from the corresponding recombinant pGEM-T clones (Accessions: KC250005, KC250006 and HF675000), sub-cloned into pDONR 221 entry vector, followed by subsequent cloning into ph7GWIWG2(II) destination vector using the LR recombination in sense and antisense orientation, using GATEWAY recombination cloning kit (Invitrogen, Carlsbad, CA, USA). The recombinant vectors were transformed into *E. coli* (DH5α), mobilized into *A*. *tumefaciens* (LBA4404), and confirmed the orientation of target genes by PCR using gene specific primers, CaMV 35S promoter and attB2, CaMV 35S terminator and attB2, *hptII* marker specific primers. Primer details are given in **Table S1**.

For *Agrobacterium* primary culture preparation, the positive clones were inoculated into 5 ml of liquid Yeast Extract Mannitol (YEM) medium (Sambrook et al., 1989) supplemented with 100 mg L^–1^ spectinomycin (Sigma Aldrich, St. Louis, Missouri, USA) and 30 mg L^–1^ rifampicin (Sigma Aldrich, St. Louis, Missouri, USA), and incubated at 28°C for 48 h at 200 rpm in an incubator shaker (New Brunswick Innova 44; Eppendorf, Hamburg, Germany). Primary culture was re-inoculated into 100 ml of antibiotic supplemented YEM medium, incubated overnight at aforesaid conditions, until the A_600_ (*Agrobacterium* suspension cells) reaches at 0.8. The culture was centrifuged for 20 min at 5,000×*g* to pellet the cells which were re-suspended in Murashige–Skoog (MS) medium (MS salt 4.41 g L^–1^, sucrose 1.5%; pH 5.4) containing 150 μM of acetosyringone and used for co-cultivation.

*O. sativa* cv. Taipei 309 was used for *Agrobacterium-* mediated transformation. The seeds were procured from Division of Genetics, ICAR-Indian Agricultural and Research Institute, New Delhi, India. Healthy seeds were de-husked and surface sterilized with 70% ethanol for 90 s, followed by washing with 50% (v/v) sodium hypochlorite solution for 20 min. Seeds were then washed with autoclaved double distilled water thrice until traces of sodium hypochlorite vanished completely. About 50 surface sterilized rice seeds were inoculated aseptically on callus induction medium (MCI) and incubated at 26 ± 2°C in dark for development of embryogenic calli. The calli were then co-cultivated by immersing them in *Agrobacterium* suspension for 20 min, excess moisture removed using a sterilized filter paper (Whatman Grade 4, Whatman International, UK), transferred on to the co-cultivation medium (MCCM, pH 5.8) containing 150 μM of acetosyringone and incubated in dark for 48 h.

After co-cultivation, the infected calli were washed with sterilized double distilled water for 25 min, containing 300 mg L^–1^ cefotaxime (HIMEDIA, Mumbai, India) and 200 mg L^–1^ ticarcillin (Sigma Aldrich, St. Louis, Missouri, USA). Calli were then transferred onto the selection medium-I (MSM I) and incubated for 15 days in dark. After primary selection, the healthy calli were transferred to fresh selection medium twice at 14 days interval for further proliferation. The newly proliferated macro-calli were grown on regeneration medium-I (MSRM-I, pH 5.8) for 7 days at 26 ± 2°C in dark and subsequently sub-cultured on fresh MSRM-I before exposing to light (light:dark photoperiod 16:8 h). Thereafter, the healthy regenerated shoots were transferred to rooting medium (RM) for root development, and later rooted plants were grown in ½ strength Yoshida’s nutrient solution (Yoshida et al., 1976) for 7 days. Plants with well-established roots were hardened in Soilrite-mix and moved to the green house for further development and seed setting. T_0_ seeds were harvested and used for further studies by raising T_1_ generation plants.

### Molecular Characterization of Transgenic Rice Plants

Genomic DNA (gDNA) was isolated from young leaf tissues of primary transformants and wild type (WT) plants using Nucleospin Plant II DNA extraction kit (Macherey-Nagel, Düren, Germany). Presence of transgenes was primarily confirmed by PCR using target genes specific primers. T_1_ generation plants were raised in autoclaved soil and DNA was isolated from the fresh leaves as stated above and PCR confirmation of the transgenes was done using different sets of primers (**Table S1**).

For determination of T-DNA integration and transgene copy number, gDNA was isolated from young leaf tissues of T_1_ of both sets of transgenics expressing *Mg-flp-1*; *Mg-flp-12* and WT plants; and Southern hybridization was performed. About 12 μg of gDNA was digested with *Sac*I (20 U μl^–1^) (New England Biolabs, Massachusetts, USA) at 37°C for 16 h. Digested DNA was then resolved on 0.8% high resolution Meta Phor agarose gel and transferred to nitrocellulose membrane (Bio-Rad, Hercules, California, USA). Probe synthesis (*Mg-flp-1*: 214 bp and *Mg-flp-12*: 299 bp), digoxigenin (DIG) labeling, hybridization, detection and blot development was carried out as described previously (Papolu et al., 2013).

Following Southern blot analysis, Northern hybridization was performed to check the presence of siRNA in the transgenics. For this, total RNA (small and large RNA in single fraction) was extracted from young leaves of T_1_ plants using NucleoSpin^®^ miRNA Kit (Macherey-Nagel, Düren, Germany). Separation of RNA, membrane transfer, probe preparation (*Mg-flp-1*: 214 bp and *Mg-flp-12*: 299 bp), DIG labeling, hybridization and detection was carried out as described earlier (Papolu et al., 2013).

### Quantification of Expression of Target Genes in Plants and Nematodes

Transgenic plants, after being confirmed by PCR and Southern blotting, from different lines of the two genes were subjected to quantitative real-time PCR (qRT-PCR) to analyze the transcript abundance of *Mg-flp-1* and *Mg-flp-12*. Total RNA was extracted from young leaves of T_1_ plants using Nucleospin plant II RNA kit (Macherey-Nagel, Düren, Germany), and assessed for quality and quantity using Nanodrop ND-1000 spectrophotometer (Thermo Scientific, Waltham, MA, USA). Approximately 500 ng of purified RNA was reverse transcribed (Superscript VILO, Invitrogen, Carlsbad, CA, USA), and qRT-PCR was carried out using gene specific primers in realplex^2^ thermal cycler (Eppendorf, Hamburg, Germany). Gene expression was normalized using *O. sativa* *18S rRNA* (Accession: AF069218). Three biological and three technical replicates were kept for each sample, and data were analyzed by 2^–ΔΔCt^ method (Livak and Schmittgen, 2001).

For analysis of expression abundance of *Mg-flp-1* and *Mg-flp-12* in the nematodes feeding on T_1_ transgenic lines, mature females were extracted from the transgenic and WT plants. RNA extraction, cDNA preparation and qRT-PCR analysis were done as described earlier (Shivakumara et al., 2019). All analyses were based on three biological and three technical replicates and nematode *18S rRNA* (Accession: HE667742) was used for normalization.

### Bioefficacy Analysis of T_1_ Transgenics Against *M. graminicola*

The T_1_ plants expressing dsRNA constructs of *Mg*-*flp-1*, *Mg-flp-12*, and the *gfp* and WT controls were initially evaluated against *M. graminicola* using Pluronic gel medium, PF-127 (Wang et al., 2009; Kumari et al., 2016). Four day-old rice seedlings (T_1_, WT and reference control plants) were used for evaluation. Approximately, 30 *M. graminicola* J2s were inoculated at the root tip of each seedling, and assays were performed as described earlier (Kumari et al., 2016). There were five replicates for each event and the parameters like total number of galls, endoparasites, number of egg masses and eggs per egg mass per plant were used for disease scoring. Nematode multiplication factor was derived as described earlier (Kumari et al., 2017).

The transgenic lines, after being evaluated on PF-127, were further screened in soil system under greenhouse conditions. Five replicates were kept for each event keeping the WT and *gfp-*control plants as reference. Twenty day-old seedlings raised singly in pots filled with autoclaved soil were inoculated at the rate of 2 J2s per g of soil and plants were grown in greenhouse as described earlier (Hatzade et al., 2020). Plants were uprooted carefully at 45 days post inoculation (dpi), roots washed free of soil and scored for total number of galls, endoparasites, number of egg masses and eggs per egg mass. Nematode multiplication factor was derived as described earlier (Kumari et al., 2017). Photographs were taken using a ZEISS SteREO Discovery V20 microscope.

### Statistical Analyses

The bioassay data were subjected to one way analysis of variance (ANOVA) using completely randomized design (CRD), and statistical significance was determined at P = 0.05 and P = 0.01. Values of mean of total replications from each treatment were taken for statistical analyses for individual assays.

## Results

### Target Identification of *Mg-flp-1* and *Mg-flp-12* for Generating HIGS Constructs

The amplified sequences of *Mg-flp*-*1* (214 bp) and *Mg-flp*-*12* (299 bp) neither showed similarity with any sequence at nr database nor showed homology with any *M. graminicola* sequence. The target dsRNA sequences were queried in dsCheck database to identify the potential off-target sites, and no exact match could be found for the processed siRNAs (19 nucleotides) in the existing database (**Figure S1**). Therefore, silencing of the aforesaid genes may not induce any off-target effects on other organisms.

RNAi Gateway vector ph7GWIWG2(II) was used to clone *Mg-flp-1*, *Mg-flp-12* and *gfp* genes flanked by attB1 and attB2 sites in sense and antisense orientation (**Figure S2**). PCR analysis confirmed the orientation of the target genes (**Figure S3**).

### Validation of RNAi Vectors of *Mg-flp-1* and *Mg-flp-12* in *O. sativa* cv. Taipei 309 Using *A. tumefaciens*

The embryogenic calli of rice cv. Taipei 309 were used for *Agrobacterium-*mediated genetic transformation with hairpin constructs of *Mg-flp-1*, *Mg-flp-12* and *gfp*, separately. *Agrobacterium*-infected calli were proliferated, histo-differentiated and transferred to MSM-I (**Table S2**). Following incubation, some calli remained healthy-creamish, whereas others turned brownish. The healthy calli were transferred to fresh MSM-II for further proliferation and later-transferred to MSRM-I to induce shoot development (**Figure S4**). The regenerated plants were hardened in soilrite-mix. A regeneration frequency of 47% was observed in the transformed calli (**Table S3**).

### Molecular Characterization of Transgenic Rice Plants Harboring RNAi Constructs

Ten primary transgenic events (T_0_) of each of *Mg-flp-1* and *Mg-flp-12* were genotyped by PCR using gene specific, CaMV 35S promoter and attB2, CaMV 35S terminator and attB2, and *hptII* primers. No amplification was observed in the WT plants while transgenic plants yielded expected amplicons (**Figures S5A, B**). Independent transformed events were confirmed by presence of transgenes conferring hygromycin resistance with an overall transformation efficiency of 6.4% (**Table S3**).

The T_1_ progeny plants were generated by selfing the selected T_0_ plants and re-validated by PCR using the aforesaid pair of primers, which showed amplification of expected fragments in all the tested lines (**Figures S6A, B**).

**Figure 1:**
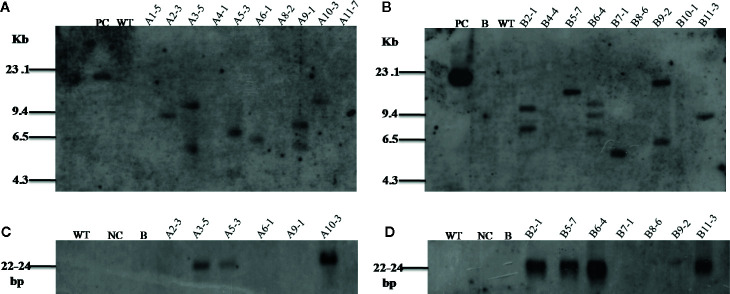
Southern and Northern blot analyses for transgenic lines harboring dsRNA constructs of *Mg-flp-1* and *Mg-flp-12*. **(A)** Southern blot analysis of *Mg-flp-1* expressing lines; A1-5, A2-3, A3-5, A4-1, A5-3, A6-1, A8-2, A9-1, A10-3, A11-7, and **(B)** *Mg-flp-*12 expressing lines; B2-1, B4-4, B5-7, B6-4, B7-1, B8-6, B9-2, B10-1, B11-3. **(C)** Northern blot analysis for *Mg-flp-1*-specific sRNA in selected transgenic lines; A2-3, A3-5, A5-3, A6-1, A9-1, A10-3, and **(D)** *Mg-flp-12*-specific sRNA in selected transgenic lines; B2-1, B5-7, B6-4, B7-1, B8-6, B9-2, B11-3. (PC, positive control; NC, negative control; B, blank; WT, wild type plant).

In order to confirm the integration and copy number of transgenes in various transgenic lines, PCR positive T_1_ plants were analyzed by Southern blot hybridization. It was found that the lines A2-3, A3-5, A5-3, A6-1, A9-1, and A10-3 for *Mg-flp-1*, and lines B2-1, B5-7, B6-4, B7-1, B9-2, and B11-3 for *Mg-flp-12* showed positive integration. WT plants were used as control (**Figures 1A, B**).

As a key component of HIGS, expression of siRNAs of *Mg-flp-1* and *Mg-flp-12* was established by Northern blot analysis in the transgenic lines. The siRNAs were detected in the representative samples of *Mg-flp-1* (A3-5, A5-3, A10-3) and *Mg-flp-12* (B2-1, B5-7, B6-4, B11-3) transgenic lines (**Figures 1C, D**), confirming the possibility for HIGS.

Transgenic plants expressing *Mg-flp-1* and *Mg-flp-12* dsRNA constructs were further confirmed at mRNA level by qRT-PCR. The results indicated variable level of expression of the transgenes in all the selected lines. The line A5-3 of *Mg-flp-1* and B5-7 of *Mg-flp-12* had the highest expression, whereas line A10-3 of *Mg-flp-1* and B8-6 of *Mg-flp-12* showed least expression (**Figure S7**). However, no expression was detected in the WT plants.

**Figure 2:**
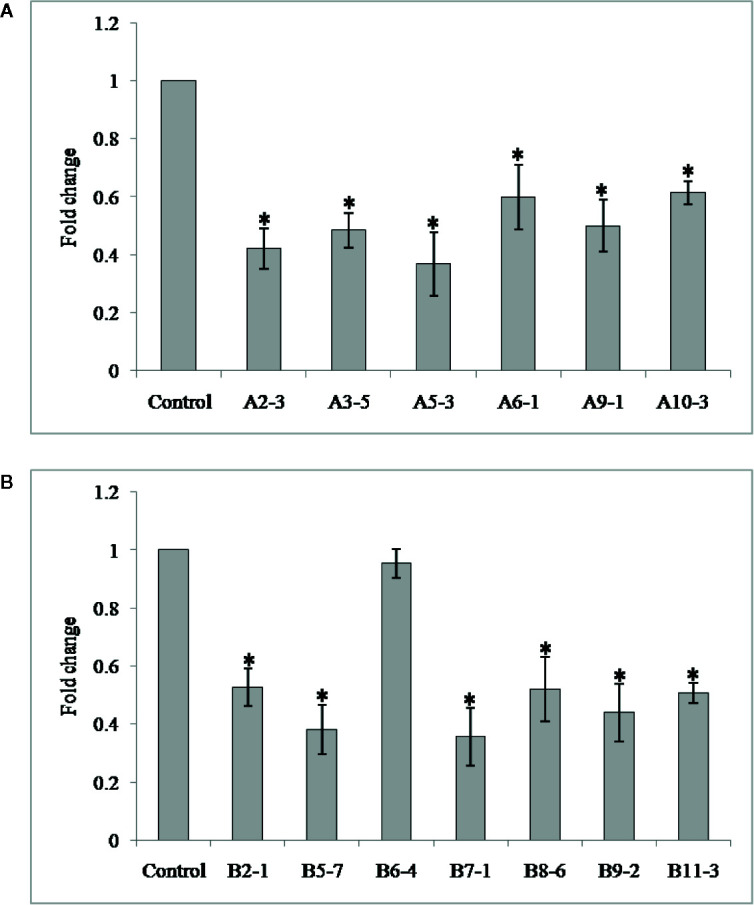
Transcript levels of *Mg-flp-1* and *Mg-flp-12* in *M. graminicola* females extracted from dsRNA expressing transgenic lines. **(A)** *Mg-flp-1* lines; A2-3, A3-5, A5-3, A6-1, A9-1, A10-3, **(B)** *Mg-flp-12* lines; B2-1, B5-7, B6-4, B7-1, B8-6, B9-2, B11-3. Expression was quantified as fold change values calculated by 2^–ΔΔCT^ method, and *18S rRNA* gene was used as reference. Each bar represents the mean ± SE of *n* = 3, and asterisks indicate significant difference at P < 0.05.

In order to investigate the effect of HIGS in suppressing target genes in nematode, qRT-PCR was performed with adult females of *M. graminicola* extracted from the transgenic plants of both genes. *Mg-flp-1* extracted females exhibited down regulation in the range of 0.63 ± 0.2–1.71 ± 0.2 fold while that of *Mg-flp-12* showed 0.04 ± 0.1–1.80 ± 0.2 fold reduction (*P* ≤0.05) (**Figure 2**).

### Bioefficacy Analysis of T_1_ Transgenics of Rice Against *M. graminicola*

**Figure 3:**
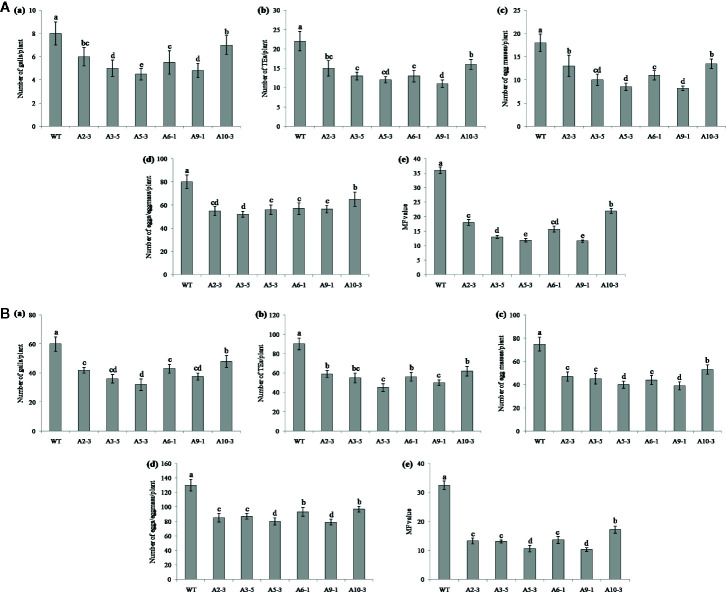
Effect of host induced silencing of *Mg-flp-1* on development and reproduction of *M. graminicola.* Performance of transgenic rice plants was assessed on **(A)** Pluronic F-127, and **(B)** soil medium. Relative number of galls (a), total number of endoparasites (b), egg masses (c), eggs per egg mass (d), and the respective multiplication factor (e) were determined in different transgenic lines (A2-3, A3-5, A5-3, A6-1, A9-1, A10-3) and wild type plants at 18 and 45 dpi. Each bar represents the mean ± SE of *n* = 5, and bars with different letters (within each parameter) denote significant difference at *P < *0.05.

**Figure 4:**
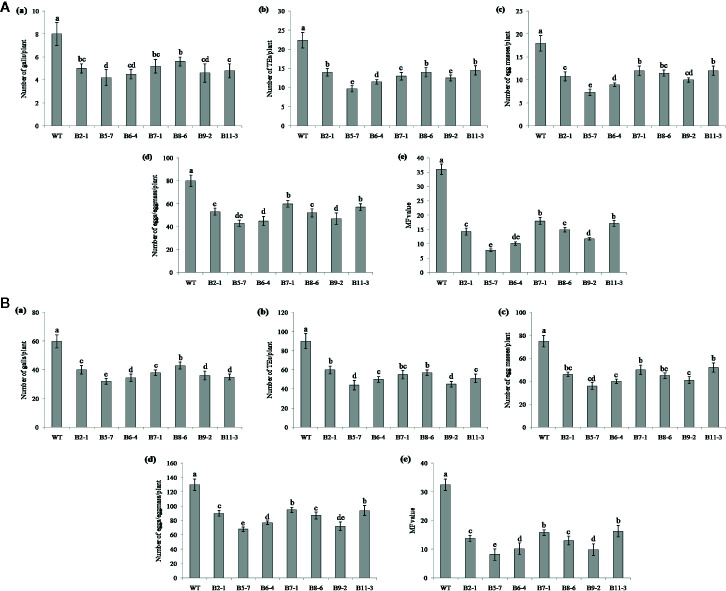
Effect of host induced silencing of *Mg-flp-12* on development and reproduction of *M. graminicola.* Performance of the transgenic rice plants was assessed on **(A)** Pluronic F-127, and **(B)** soil medium. Relative number of galls (a), total number of endoparasites (b), egg masses (c), eggs per egg mass (d), and the respective multiplication factor (e) were determined in different transgenic lines (B2-1, B5-7, B6-4, B7-1, B8-6, B9-2, B11-3) and WT plants at 18 and 45 dpi. Each bar represents the mean ± SE of *n* = 5, and bars with different letters (within each parameter) denote significant difference at *P < *0.05.

**Figure 5:**
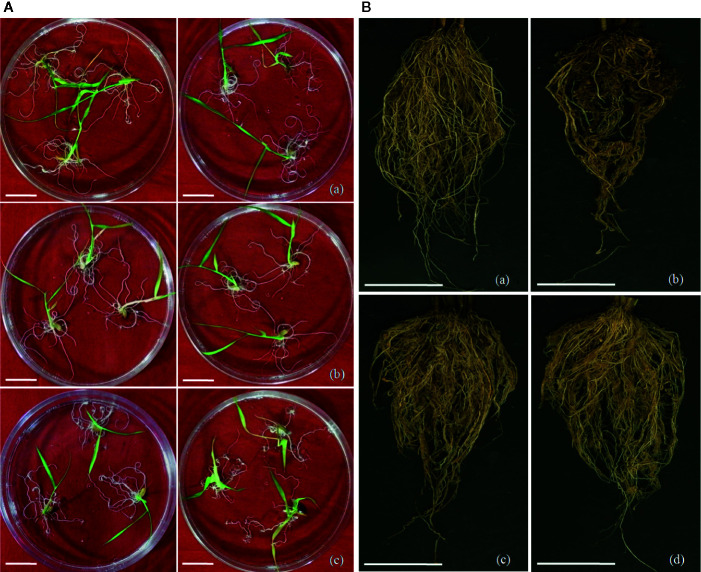
Screening of transgenic rice lines for nematode infection. **(A)** Screening of *M. graminicola* infected plants in PF-127 medium at 18 dpi; representative nematode infected plants of (a) *Mg-flp-1* transgenic lines, (b) *Mg-flp-12* transgenic lines, (c) wild type plants. Typical hook-like galls on roots with greater number of them in WT compared to transgenic lines; scale bar = 0.5 cm, **(B)** Comparison of *M. graminicola* infection in transgenic plant roots and WT plants at 45 dpi; representative roots of (a) healthy WT plants, (b) nematode infected WT plants, (c) nematode infected *Mg-flp-1* transgenics, and (d) nematode infected *Mg-flp-12* transgenics. Intensity of galling was comparatively higher in WT plants than the transgenics; scale bar = 5 cm.

Performance of the transgenic plants was initially assessed against *M. graminicola* on Pluronic F-127 medium. Number of galls, endoparasites and egg masses developed per plant, and eggs per egg masses were counted at 18 dpi. The results showed reduction in average galling by 13‒44% for *Mg-flp-1* (**Figure 3A**) and 30‒48% for *Mg-flp-12* in different transgenic lines (**Figure 4A**). This observation was corroborated with reduced number of endoparasites which was 28‒46% for *Mg-flp-1* and 36‒57% for *Mg-flp-12* transgenic lines. Likewise, a significant reduction was seen in number of egg masses (25–55%) and eggs per egg mass (19–35%) in *Mg-flp-1* transgenics. Similarly, *Mg-flp-12* silencing decreased number of egg masses by 34–60% and eggs per egg mass by 29–47%. Finally, derived multiplication factor was reduced in *Mg-flp-1* and *Mg-flp-12* silenced plants by 40‒68% and 53‒78%, respectively when compared to the WT and negative control plants (**Figure 5A**).

The independent T_1_ lines harboring RNAi constructs were further assessed for performance in soil by inoculating 20-day old plants with freshly hatched J2s. The plants were harvested after completion of two successive life cycles of nematode. The results demonstrated that transgenic plants of different lines showed significant reduction (*P* ≤0.05) in gall number compared to WT plants and control plants. Line A10-3 of *Mg-flp-1* (**Figure 3B**) and B8-6 of *Mg-flp-12* (**Figure 4B**) showed highest number of galls but also showed significant reduction in total endoparasites (31–37%), egg masses (30–40%) and eggs per egg mass (25–33%). The derived multiplication factor was found to be reduced by 47–60% (**Figure 5B**).

## Discussion

In the present study, we have evaluated HIGS of two FMRFamide-like peptide (FLP) genes of *M. graminicola*, *Mg*-*flp-1* and *Mg*-*flp-12* in rice. Nematode neuropeptides, especially the FLPs are associated with numerous physiological functions including host recognition, feeding, sensory perception, navigation, reproduction and parasitism (Kimber et al., 2007; Morris et al., 2017; Warnock et al., 2017). Hence, these neuropeptides could be potential targets for designing safe and specific nematode management schedule (Peymen et al., 2014; Warnock et al., 2017). Several FLPs have been identified from the PPNs, and FMRFamide-like immune reactivity has also been observed in the nervous systems of some PPNs like *G. pallida* and *G. rostochiensis* (Kimber et al., 2001). To date, 19 *flp* genes have been identified in the most notorious nematode species *M. incognita*, and HIGS of *flp-14* and *flp-18* in tobacco provided significant reduction in reproductive potential and parasitism of the nematode (Papolu et al., 2013). Rice production is largely affected by *M. graminicola* (Jones et al., 2013), and earlier observations demonstrated that *in vitro* silencing of nine *flp* genes (*flp-1*, *flp-3*, *flp-6*, *flp-7*, *flp-11*, *flp-12*, *flp-14*, *flp-16* and *flp-18*) in *M. graminicola* J2s significantly retarded their penetration and reproduction potential while infecting rice (Kumari et al., 2017). Further, results involving *G. pallida*, *G. rostochiensis*, *Heterodera glycies*, *H. schachtii*, *M. incognita*, *M. javanica*, *Pratylenchus* spp., etc. suggest that HIGS of parasitism genes provide a better platform for nematode management and also strengthens the findings of *in vitro* RNAi (Urwin et al., 2002; Chen and Roberts, 2003; Vanholme et al., 2004; Dubreuil et al., 2007; Adam et al., 2008; Bakhetia et al., 2008; Tan et al., 2013; Chaudhary et al., 2019; Shivakumara et al., 2019). In this regard, the present study convincingly supports the earlier findings of Kumari et al. (2017), and also validates the efficiency of *M. graminicola flp-1* and *flp-12*, as potential targets for nematode management through HIGS in Asian rice *O. sativa*.

Molecular analyses by PCR and Southern hybridization revealed the insertion, integration and inheritance of the T-DNA harboring dsRNA constructs of *Mg-flp-1* and *Mg-flp-12* independently in most of the transformed lines. However, the expression of hpRNA coding transgenes quantified by qRT-PCR was found to be variable among different lines. This suggests that the transgenes were randomly integrated at diverse transcriptionally active sites in the rice genome. Additionally, the nematode gene sequences showed no similarity or homology with any short stretches of similar sequences in rice genome, which reduces the risk of any non-target effect.

The results of nematode bioassays revealed significant reduction in development and reproduction in most of the transgenics lines, containing dsRNA construct of *Mg-flp-1* and *Mg-flp-12*, when compared to the WT plants. Simultaneously, galling and multiplication factor also declined significantly in the transgenic plants. No apparent morphological variation was observed in transgenic lines compared to the WT plants, indicating the focused RNAi effect. In addition, the detection of target gene specific siRNAs in transgenic lines provided authentic evidence for the HIGS of *Mg-flp-1* and *Mg-flp-12* genes, which resulted in reduced virulence and reproduction of the nematode species. A previous study has showed that *in vitro* silencing of both these genes reduced penetration and infection of the *M. graminicola* juveniles (Kumari et al., 2017). This finding can be corroborated with some other studies (Kimber et al., 2007; Papolu et al., 2013), where silencing of *flp* genes also showed similar results. dsRNA mediated silencing of *flp-1*, *flp-6*, *flp-12*, *flp-14* and *flp-18* genes in *G. pallida*, led to aberrant behavioral phenotypes (Kimber et al., 2007). Host induced silencing of *flp-14* and *flp-18* in tobacco affected development of *M. incognita* (Papolu et al., 2013). In *B.* *xylophilus*, *flp*-*4* and *flp*-*18* might coordinate with NPR-4 receptor, and *flp-3*, *flp-18*, *flp-7* and *flp*-*11* activate NPR-10 and FRPR-3 receptors, thus forming an integral part of complex neuronal network controlling nematode movement through motor and sensory neurons (Kimber et al., 2007). The reduced development, reproduction and parasitism as a result of silencing *Mg-flp-1* and *Mg-flp-12* might also involve a complex neuromuscular system in *M. graminicola* that affects the rice-nematode interaction. Studies with *Ascaris suum* revealed that silencing of *flp-14* and 15 other *flps* inhibit nematode ovijection by shortening oviduct length, relaxing the circular muscles and cessation of contractile activity simultaneously (Moffett et al., 2003). Similar physiological anomalies might take place upon silencing of *Mg-flp-1* and *Mg-flp-12*, which possibly result in reduction in nematode’s egg laying capacity. Similar findings were also recorded in the model nematode *Caenorhabditis elegans*, where loss of function mutants of *flp-1* showed altered locomotion and egg laying (Chang et al., 2015).

In order to assess the HIGS of *Mg-flp-1* and *Mg-flp-12*, the adult females were extracted from the T_1_ transgenic lines and WT plants and qRT-PCR was performed to assess the transcript abundance. A significant reduction in gene expression (up to 1.8 folds) was observed in the nematode females extracted from the transformed lines. These findings unequivocally support that HIGS of both *flp* genes in rice could effectively control the RKN species *M. graminicola* independently, and are adequately substantiated by the inclusion of negative controls during *in planta* RNAi studies.

Although there are several reports of efficacy of *flp* genes disrupting the neuromotor functions in nematodes, this is an established report to demonstrate the effectiveness of *Mg-flp-1* and *Mg-flp-12* genes in the management of *M. graminicola* using HIGS in rice. The significant reduction of nematode multiplication factor in the transgenic plants has proved the potential of RNAi silencing of both genes. Based on the environmental effects, agronomic conditions and biology of the nematodes, absolute resistance is very hard to achieve against these pests (Fuller et al., 2008; Rosso et al., 2009). In this direction, *Mg-flp-1* and *Mg-flp-12* genes would be highly effective to withstand the initial population toll in the field, and will also help bringing down the population build up that will help in reducing the initial nematode pressure in soil for successive crop(s). Thus the early crop growth won’t get affected. Further, sustainable management can also be achieved by pyramiding few promising *flp* genes together to efficiently interfere different physiological processes required for successful nematode parasitism.

## Data Availability Statement

All datasets generated for this study are included in the article/**Supplementary Material**.

## Author Contributions

UR conceived, designed, and supervised the experiments. AH and CK performed the experiments. DS help in plant transformation. AH analyzed the data, and wrote the draft of the manuscript. UR, VP, and VC reviewed and wrote the final draft of the manuscript. All authors contributed to the article and approved the submitted version.,

## Conflict of Interest

The authors declare that the research was conducted in the absence of any commercial or financial relationships that could be construed as a potential conflict of interest.

## Abbreviations

FLPs, FMRFamide-like peptides; HIGS, Host-induced gene silencing; dsRNA, Double-stranded RNA; hpRNA, Hairpin RNA; PPNs, Plant-parasitic nematodes; RKN, Root-knot nematode; MF, Multiplication factor.
