# Supplementary material for: Host-Induced Silencing of FMRFamide-Like Peptide Genes, flp-1 and flp-12, in Rice Impairs Reproductive Fitness of the Root-Knot Nematode Meloidogyne graminicola
Source: Front Plant Sci. 2020 Jul 17;11:894. doi: 10.3389/fpls.2020.00894 (PMC7379849; doi:10.3389/fpls.2020.00894)
Supplement: Supplementary file 1 [file DataSheet_1.docx]

SUPPLEMENTARY / SUPPORTING MATERIAL

**FIGURE S1 Target dsRNA sequences were investigated in dsCheck database to identify the potential off-target sites. (A)** *Mg-flp-1* and *Mg-flp-12* with *C. elegance*, **(B)** *Mg-flp-1* and *Mg-flp-12* with *Oryza Sativa*. No significant matches/hits were detected at specified threshold for the processed siRNAs (19 nucleotides) in the existing database. mis=0 describe the total hits with complete matches; mis=1 describe the total hits with the one mismatches; mis= 2 describe total hits with the 2 mismatches.

**FIGURE S2** **T-DNA region of binary vector used for gene silencing in rice,** **Taipei 309.** (**A**) pH7GWIWG2(II)–Mg-flp-1, (**B**) pH7GWIWG2(II)–Mg-flp-12*,* **(C)** pH7GWIWG2(II)–gfp. Inverted repeats of a 214 bp of *Mg-flp-1* and 299 bp of *Mg-flp-12* as well as 375 bp of *gfp* fragments were cloned (separately). RB – T-DNA right border, LB – left border, *hpt* – hygromycin phosphotransferase gene, P35S – Cauliflower mosaic virus (CaMV) 35S promoter, T35S – CaMV 35S terminator, *Mg-flp-1* and *Mg-flp-12* – *M. graminicola* FMRFamide like peptides (FLPs), *gfp –* green fluorescent protein, attB1 and attB2 – LR reaction sites.

**FIGURE S3 Cloning confirmation of *Mg-flp-1* and *Mg-flp-12* into RNAi constructs pH7GWIWG2(II). (A)** *Mg-flp-1,* (**B**) *Mg-flp-*12. PCR confirmation using four sets of primers specific to target genes; CaMV 35S promoter; CaMV 35S terminator; and *hptII* to confirm the orientation of the target genes and selectable marker respectively. The amplified products were resolved on 1% agarose gel. M1 – 100 bp DNA ladder; M2 – 1 kb DNA ladder; PC – gene specific positive control; NC – negative control; 1– target gene; 2 – CaMV 35S promoter; 3 – CaMV 35S terminator; 4 – *hptII* specific amplification.

**FIGURE S4 *Agrobacterium-* mediated transformation of rice with RNAi gene constructs.** (**A**) Callus initiation of the explants, (**B–C)** First and second round of selection on medium containing hygromycin, **(D)** Callus differentiation, (**E**) Microcalli on regeneration-I, (**F**) Shoot induction in regeneration-II.

**FIGURE S5A PCR confirmation of *Mg-flp-1* transformed rice (T_0_). (A)** Amplification of the target gene using gene specific primers, (**B)** Amplification of sense strand using primers 35S promoter forward and *attB2* reverse, (**C)** Amplification of the antisense strand using primers 35S terminator forward and *attB2* reverse, (**D)** Amplification of *hptII* gene. M1 – 100 bp DNA ladder; M2 – 1 kb DNA ladder; PC – positive control; WT – wild type plant; A1 to A11 – independent T_0_ events.

**FIGURE S5B PCR confirmation of *Mg-flp-12* transformed rice (T_0_). (A)** Amplification of the target gene using gene specific primers, (**B)** Amplification of sense strand using primers 35S promoter forward and *attB2* reverse, (**C)** Amplification of the antisense strand using primers 35S terminator forward and *attB2* reverse, (**D)** Amplification of *hptII* gene. M1 – 100 bp DNA ladder; M2 – 1 kb DNA ladder; PC – positive control; WT – wild type plant; B1 to B11 – independent T_0_ events.

**FIGURE S6A PCR confirmation of *Mg-flp-1* in T_1_ generation transgenic lines. (A)** Amplification of the target gene using gene specific primers, (**B)** Amplification of sense strand using primers 35S promoter forward and *attB2* reverse, (**C)** Amplification of the antisense strand using primers 35S terminator forward and *attB2* reverse, (**D)** Amplification of *hptII* gene. M1 – 100 bp DNA ladder; M2 – 1 kb DNA ladder; PC – positive control; WT – wild type plant; A1-5 to A11-7 – T_1_ lines.

**FIGURE S6B PCR confirmation of *Mg-flp-12* in T_1_ generation transgenic lines. (A)** Amplification of the target gene using gene specific primers, (**B)** Amplification of sense strand using primers 35S promoter forward and *attB2* reverse, (**C)** Amplification of the antisense strand using primers 35S terminator forward and *attB2* reverse, (**D)** Amplification of *hptII* gene. M1 – 100 bp DNA ladder; M2 – 1 kb DNA ladder; PC – positive control; WT – wild type plant; B1-3 to B11-3 – T_1_ lines.

**FIGURE** **S7** **Expression analysis of target genes T_1_ generation in transgenic lines. (A)** *Mg-flp-1* expressing lines; A2-3, A3-5, A5-3, A6-1, A9-1, A10-3, **(B)** *Mg-flp-12* expressing lines; B2-1, B5-7, B6-4, B7-1, B8-6, B9-2, B11-3. ∆Ct values were calculated using difference in the Ct mean of target gene and reference gene (*Os* *18S rRNA*). Each bar represents the mean ± SE of *n* = 3, and asterisks indicate significant difference at P < 0.05. Higher ∆Ct values specify the lower expression of transgene in the corresponding lines.


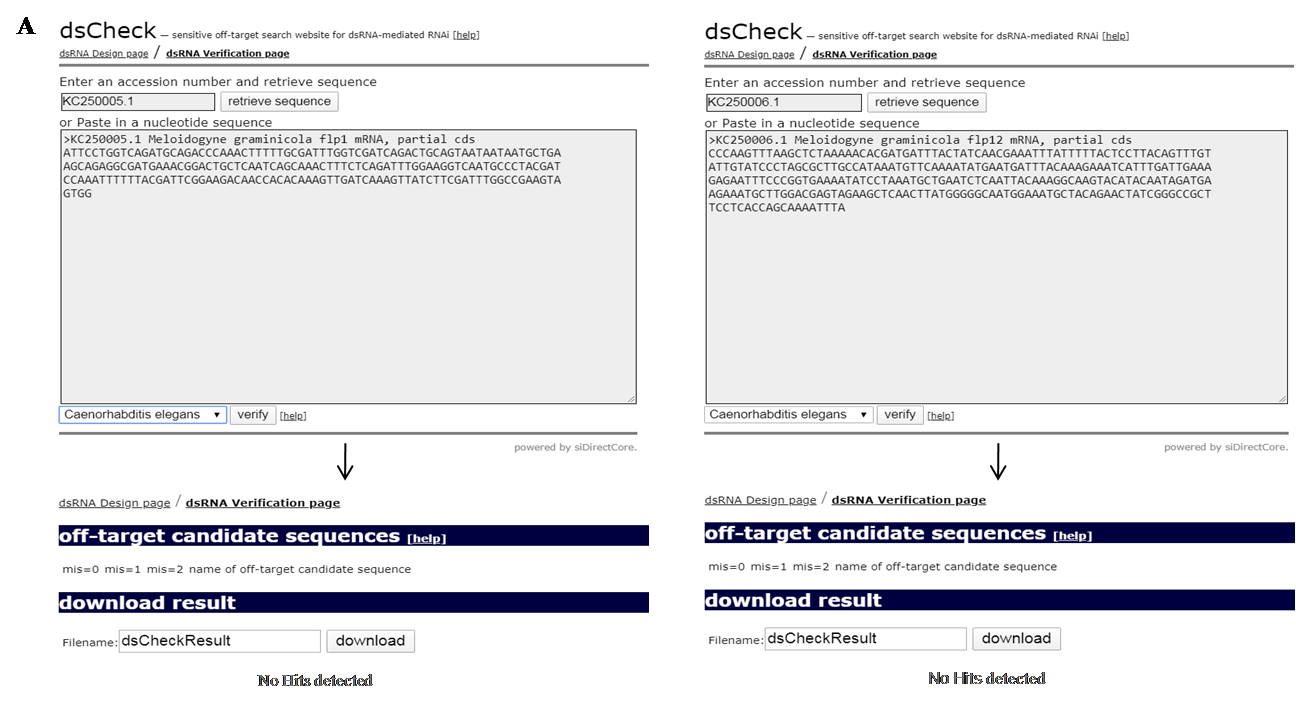


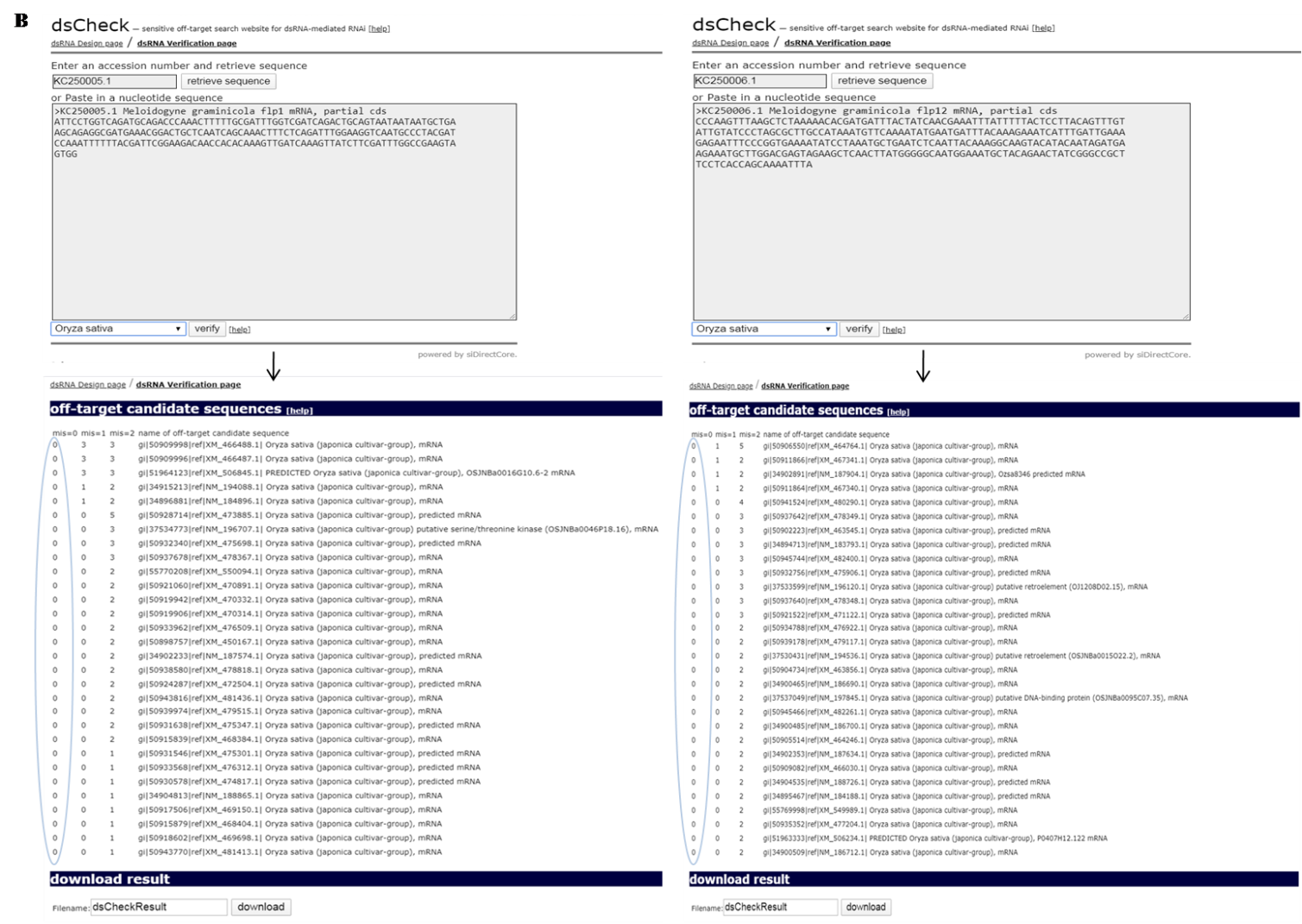


**FIGURE S1**


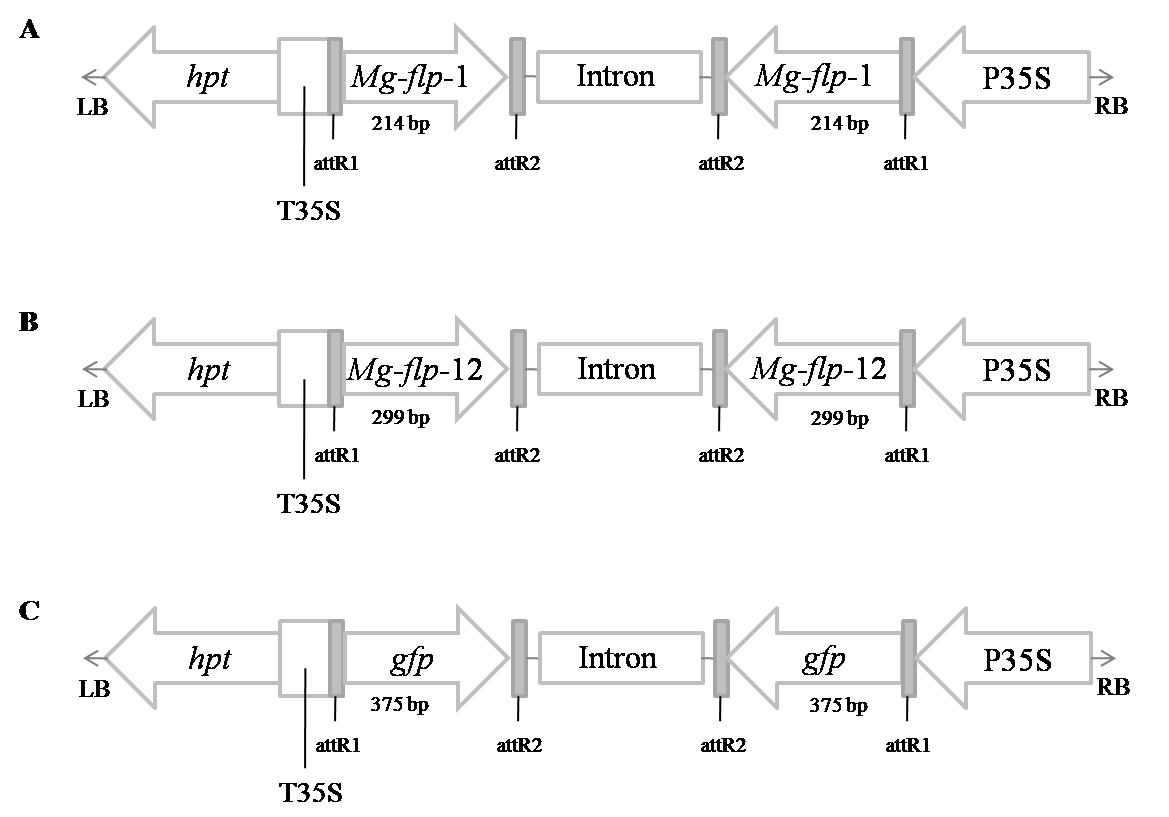


**FIGURE S2**


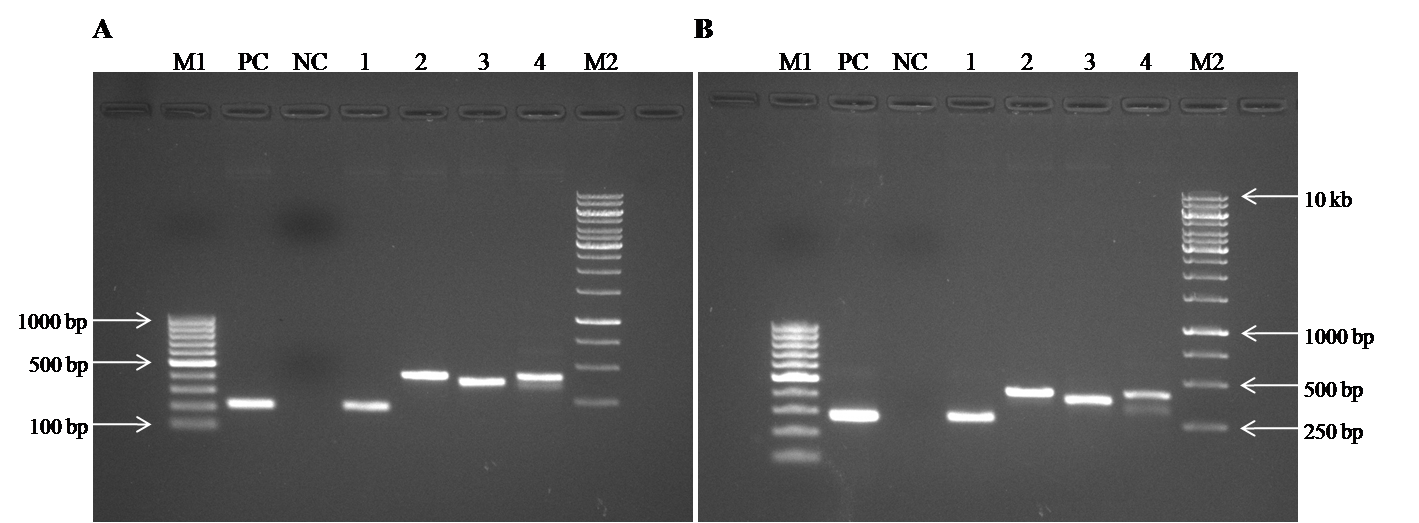


**FIGURE S3**


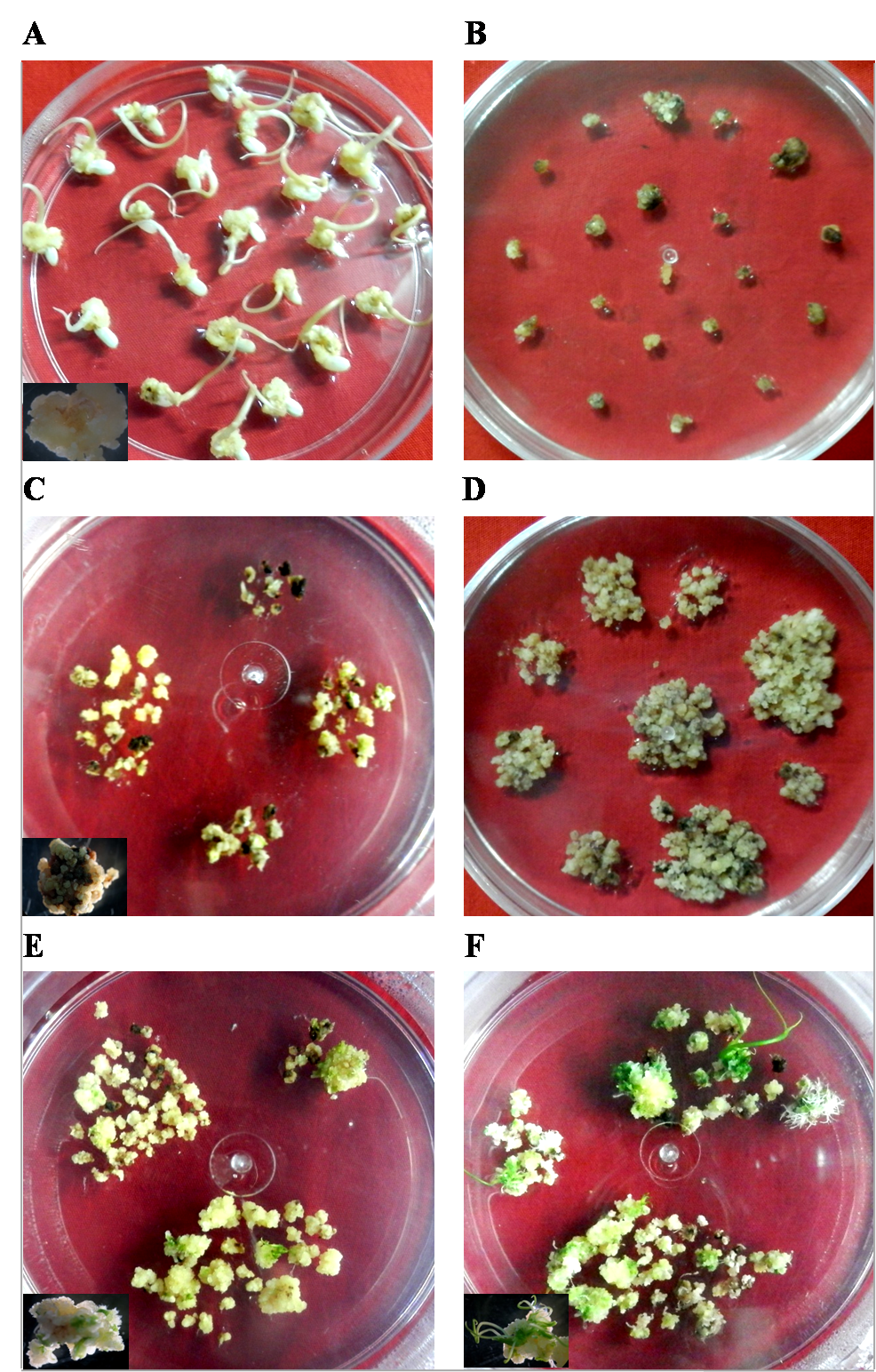


**FIGURE S4**


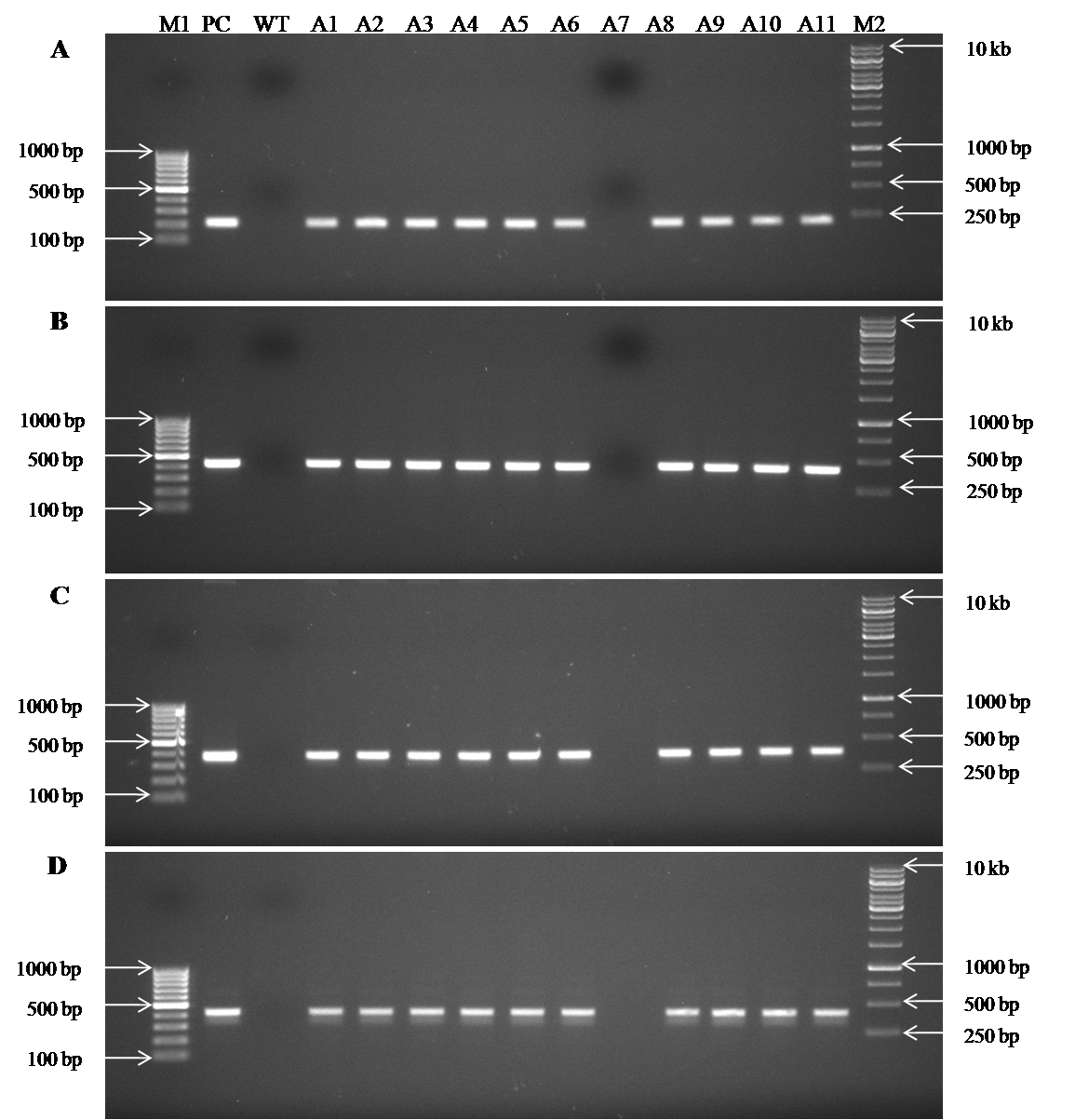


**FIGURE S5A**


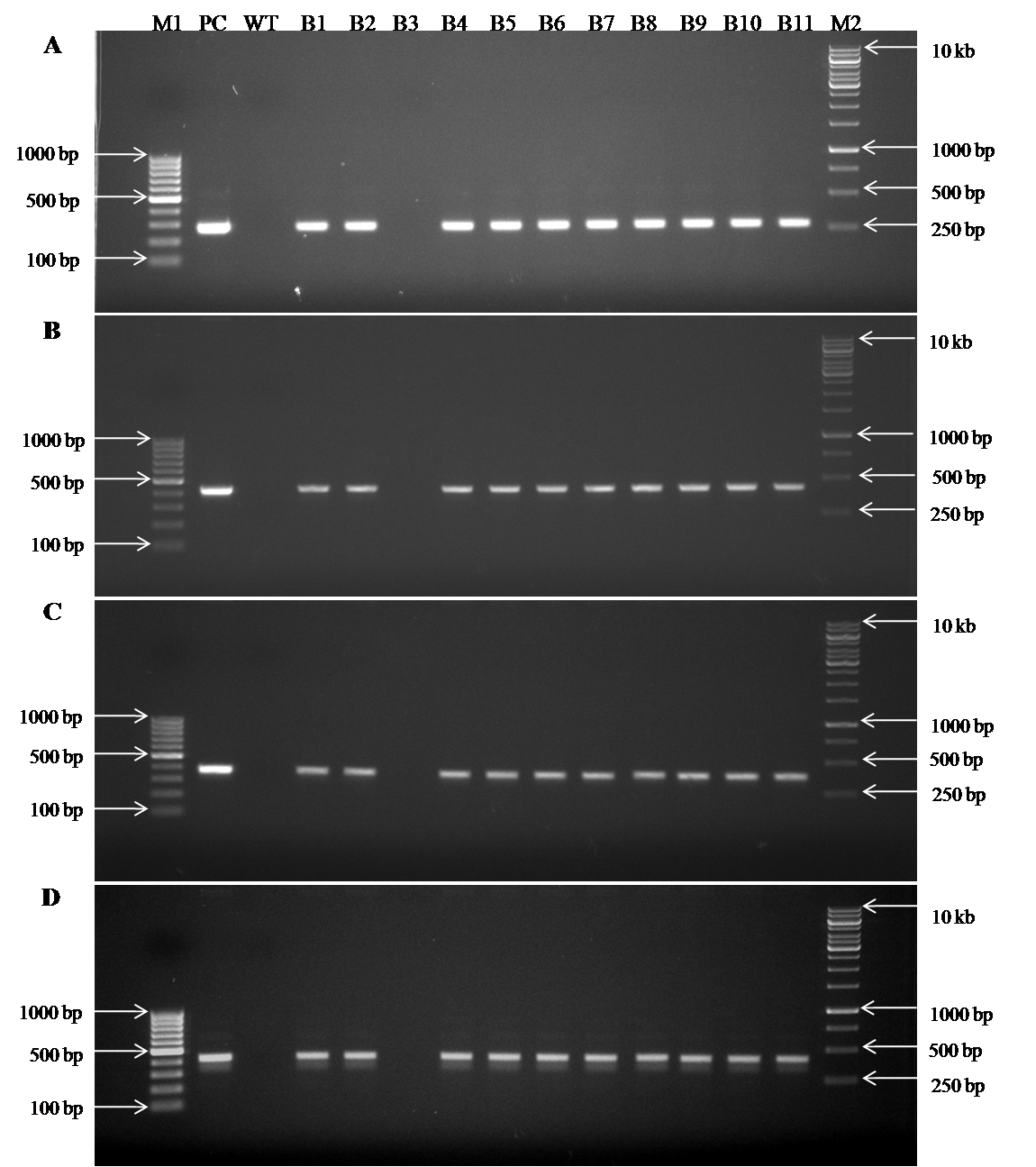


**FIGURE S5B**


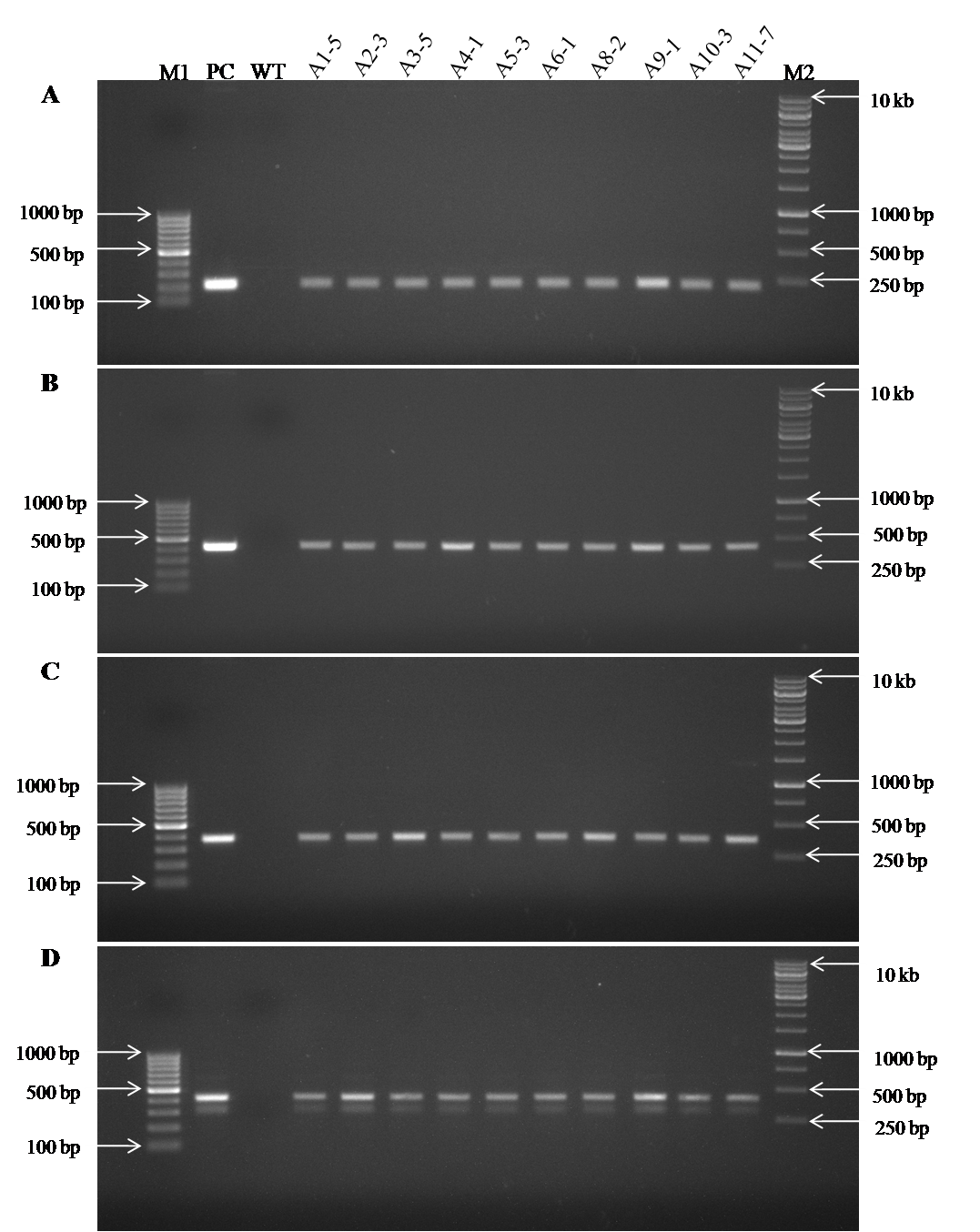


**FIGURE S6A**


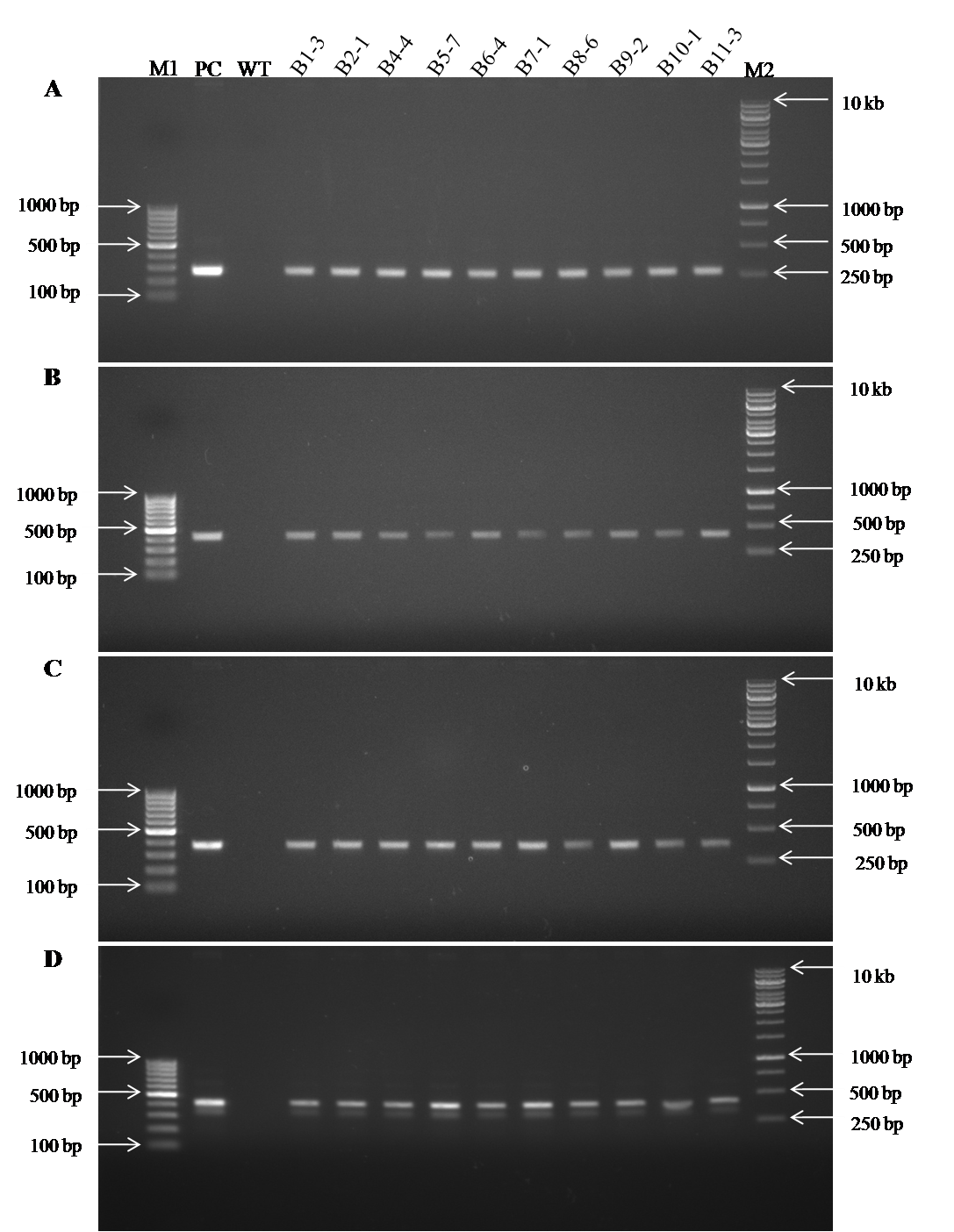


**FIGURE S6B**

**
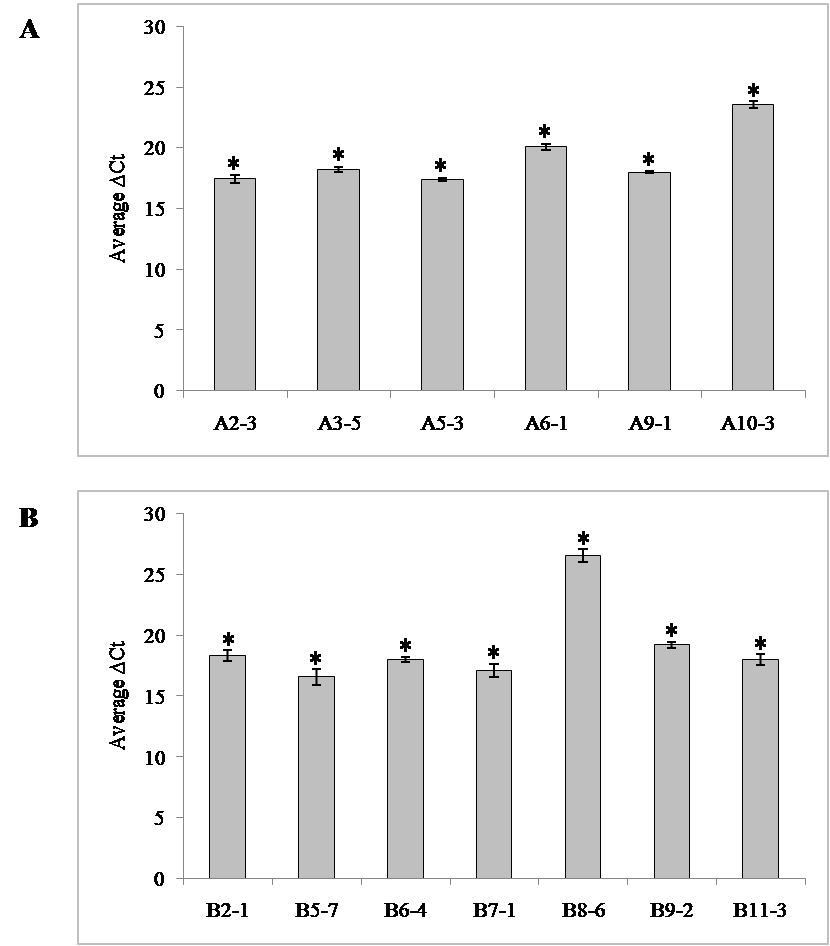
**

**FIGURE S7**

**TABLE S1 List of primers and probes used for cloning, PCR amplification, Southern and northern hybridization.**

| **Name** | **Sequence (5’ --- 3’)** |
| --- | --- |
| GV–Mg-flp-1 F | GGGGACAAGTTTGTACAAAAAAGCAGGCTATTCCTGGTCAGATGCAGACCCAA3 |
| GV–Mg-flp-1 R | GGGGACCACTTTGTACAAGAAAGCTGGGTCCACTACTTCGGCCAAATCGAAGA |
| GV–Mg-flp-12 F | GGGGACAAGTTTGTACAAAAAAGCAGGCTCCCAAGTTTGAGCTCTAAAAACAC |
| GV–Mg-flp-12 R | GGGGACCACTTTGTACAAGAAAGCTGGGTTCATCGTCCAAATCGAATGA |
| GV–GFP- F | GGGGACAAGTTTGTACAAAAAAGCAGGCTAGCGGCACGACTTCTTCA |
| GV–GFP- R | GGGGACCACTTTGTACAAGAAAGCTGGGTGTGTGGACAGGTAATGGTTGT |
| CaMV35S Promoter | TCCTTCGCAAGACCCTTC |
| CaMV35S Terminator | CCTTATCTGGGAACTACTCACAC |
| attB1 | GGGGACAAGTTTGTACAAAAAAGCAGGCT |
| attB2 | GGGGACCACTTTGTACAAGAAAGCTGGGT |
| hptII F | CGAAATTGCCGTCAACCAAGCTCT |
| hptII R | CATGGCGTGATTTCATATGCGCGA |
| RT–Mg-flp-1 F | TTTGGGTGCTACAAGTGCCAACAG |
| RT–Mg-flp-1 R | TCCTTCTTCGTTTGAGGCAGCAGA |
| RT–Mg-flp-12 F | TGAGGAAGCGGCCCGATAGTTCTT |
| RT–Mg-flp-12 R | GGATGAAGAAATGCTTGGACGAGT |
| Mg-18S rRNA F | CAACGTGCTTGTCCTACCCTGAA |
| Mg-18S rRNA R | TGTGTACAAAGGGCAGGGACGTA |
| Os-18S rRNA F | CGCGCAAATTACCCAATCCTGACA |
| Os-18S rRNA R | TCCCGAAGGCCAACGTAAATAGGA |

**TABLE S2** **Components used in media for the modified *O. sativa* cv. Taipei 309 transformation**

| **Medium** | **Compositions** |
| --- | --- |
| MCI | 4.14 g L^–1^ MS Salts with Vitamins, 0.2 g L^–1^ Casein enzymatic hydrolysate, 0.5 g L^–1^ L-Proline, 2 mg L^–1^ 2,4-Dichlorophenoxyacetic acid, 3% Sucrose, 0.4% Phytogel (pH 5.8) |
| MCCM | 4.14 g L^–1^ MS Salts with Vitamins, 0.2 g L^–1^ Casein enzymatic hydrolysate, 0.5 g L^–1^ L-proline, 2 mg L^–1^ 2,4-Dichlorophenoxyacetic acid, 3% Sucrose, 0.4% Phytogel, 150 µM acetosyringone (pH 5.8) |
| MSM I | 4.14 g L^–1^ MS Salts with Vitamins, 0.2 g L^–1^ Casein enzymatic hydrolysate, 0.5 g L^–1^ L-proline, 2 mg L^–1^ 2,4-Dichlorophenoxyacetic acid, 3% Sucrose, 0.4% Phytogel, 300 mg L^–1^ Cefotaxime, 200 mg L^–1^ Timentin, 50 mg L^–1^ Hygromycin (pH 5.8) |
| MSM II | 4.14 g L^–1^ MS Salts with Vitamins, 0.2 g L^–1^ Casein enzymatic hydrolysate, 0.5 g L^–1^ L-proline, 2 mg L^–1^ 2,4-Dichlorophenoxyacetic acid, 3% Sucrose, 0.4% Phytogel, 300 mg L^–1^ Cefotaxime, 200 mg L^–1^ Timentin, 35 mg L^–1^ Hygromycin (pH 5.8) |
| MSRM I | 4.14 g L^–1^ MS Salts with Vitamins, 3 mg L^–1^ 6-Benzylaminopurine, 1.5 mg L^–1^ 1-Naphthaleneacetic acid, 1 mg L^–1^ Kinetin, 3% Sucrose, 0.4% Phytogel, 300 mg L^–1^ Cefotaxime, 35 mg L^–1^ Hygromycin (pH 5.8) |
| RM | 4.14 g L^–1^ MS Salts with Vitamins, 1 mg L^–1^ 1-Naphthaleneacetic acid, 3% Sucrose, 0.4% Phytogel (pH 5.8) |

MCI– callus induction medium, MCCM– co-cultivation medium, MSM I– selection medium-I, MSM II– selection medium-II, MSRM I– regeneration medium-I, RM– rooting medium

**TABLE S3** **Transformation efficiency of *O. sativa* cv. Taipei 309 subjected to hygromycin selection**

| Experiment Number | Number of explants infected | Explants in Selection  (% of regeneration efficiency ^a^) | Number of hygromycin  resistant plants in  T_0_ generation  (PCR ^+ve^) | Final transformation  efficiency^b^ (%) |
| --- | --- | --- | --- | --- |
| 1 | 50 | 24 | 04 | 8.0 |
| 2 | 42 | 17 | 02 | 4.7 |
| 3 | 45 | 20 | 02 | 4.4 |
| 4 | 39 | 20 | 03 | 7.6 |
| 5 | 43 | 22 | 03 | 7.0 |
|  |  |  | **Mean Transformation efficiency**  **Mean Regeneration efficiency** | **6.4**  **47.0** |

^a^ Regeneration efficiency = (Number of explants regenerated on selection pressure / Number of explants infected) X 100

^b^ Transformation efficiency = (Number of PCR ^+ve^ T_0_ events / Number of explants infected) X 100
